# Donanemab (LY3002813) Phase 1b Study in Alzheimer's Disease: Rapid and Sustained Reduction of Brain Amyloid Measured by Florbetapir F18 Imaging

**DOI:** 10.14283/jpad.2021.56

**Published:** 2021-09-21

**Authors:** S.L. Lowe, C. Duggan Evans, S. Shcherbinin, Y.-J. Cheng, B.A. Willis, I. Gueorguieva, A.C. Lo, A.S. Fleisher, J.L. Dage, P. Ardayfio, G. Aguiar, M. Ishibai, G. Takaichi, L. Chua, G. Mullins, John R. Sims, AACD Investigators

**Affiliations:** 1 Eli Lilly and Company, Lilly Singapore, Singapore, Singapore; 2 Eli Lilly and Company, Lilly Corporate Center DC 1532, 46285, Indianapolis, IN, USA; 3 Eli Lilly and Company, Bracknell, UK; 4 Stark Neurosciences Research Institute, Indiana University School of Medicine, Indianapolis, IN, USA; 5 Eli Lilly Japan, K.K., Kobe, Japan

**Keywords:** Alzheimer's disease, amyloid plaque, donanemab, florbetapir PET, immunogenicity

## Abstract

**Background:**

Donanemab (LY3002813) is an IgG1 antibody directed at an N-terminal pyroglutamate of amyloid beta epitope that is present only in brain amyloid plaques.

**Objectives:**

To assess effects of donanemab on brain amyloid plaque load after single and multiple intravenous doses, as well as pharmacokinetics, safety/tolerability, and immunogenicity.

**Design:**

Phase 1b, investigator- and patient-blind, randomized, placebo-controlled study.

**Setting:**

Patients recruited at clinical research sites in the United States and Japan.

**Participants:**

61 amyloid plaque-positive patients with mild cognitive impairment due to Alzheimer's disease and mild-to-moderate Alzheimer's disease dementia.

**Intervention:**

Six cohorts were dosed with donanemab: single dose 10-, 20- or 40- mg/kg (N = 18), multiple doses of 10-mg/kg every 2 weeks for 24 weeks (N = 10), and 10- or 20-mg/kg every 4 weeks for 72 weeks (N=18) or placebo (N = 15).

**Measurements:**

Brain amyloid plaque load, using florbetapir positron emission tomography, was assessed up to 72 weeks. Safety was evaluated by occurrence of adverse events, magnetic resonance imaging, electrocardiogram, vital signs, laboratory testing, neurological monitoring, and immunogenicity.

**Results:**

Treatment with donanemab resulted in rapid reduction of amyloid, even after a single dose. By 24 weeks, amyloid positron emission tomography mean changes from baseline for single donanemab doses in Centiloids were: −16.5 (standard error 11.22) 10-mg/kg intravenous; 40.0 (standard error 11.23) 20 mg/kg intravenous; and −49.6 (standard error 15.10) 40-mg/kg intravenous. Mean reduction of amyloid plaque in multiple dose cohorts by 24 weeks in Centiloids were: 55.8 (standard error 9.51) 10-mg/kg every 2 weeks; −50.2 (standard error 10.54) 10-mg/kg every 4 weeks; and −58.4 (standard error 9.66) 20-mg/kg every 4 weeks. Amyloid on average remained below baseline levels up to 72 weeks after a single dose of donanemab. Repeated dosing resulted in continued florbetapir positron emission tomography reductions over time compared to single dosing with 6 out of 28 patients attaining complete amyloid clearance within 24 weeks. Within these, 5 out of 10 patients in the 20 mg/kg every 4 weeks cohort attained complete amyloid clearance within 36 weeks. When dosing with donanemab was stopped after 24 weeks of repeat dosing in the 10 mg every 2 weeks cohort, florbetapir positron emission tomography reductions were sustained up to 72 weeks. For the single dose cohorts on day 1, dose proportional increases in donanemab pharmacokinetics were observed from 10 to 40 mg/kg. Dose proportional increases in pharmacokinetics were also observed at steady state with the multiple dose cohorts. Donanemab clearance was comparable across the dose levels. Mean donanemab elimination-halflife following 20 mg/kg single dose was 9.3 days with range of 5.6 to 16.2 days. Greater than 90% of patients had positive treatment-emergent antidrug antibodies with donanemab. However, overall, the treatment-emergent antidrug antibodies did not have a significant impact on pharmacokinetics. Donanemab was generally well tolerated. Amongst the 46 participants treated with donanemab, the following amyloid-related imaging abnormalities, common to the drug class, were observed: 12 vasogenic cerebral edema events (12 [19.7%] patients), 10 cerebral microhemorrhage events (6 [13.0%] patients), and 2 superficial siderosis events (2 [4.3%] patients).

**Conclusions:**

Single and multiple doses of donanemab demonstrated a rapid, robust, and sustained reduction up to 72 weeks in brain amyloid plaque despite treatment-emergent antidrug antibodies detected in most patients. Amyloid-related imaging abnormalities were the most common treatment-emergent event.

## Introduction

**T**he deposition of amyloid-beta peptide (Aβ) is essential to the pathophysiology and progression of Alzheimer's disease (AD) (1), and thereby has led to the discovery and development of active and passive immunotherapies with mechanisms of action that reduce Aβ accumulation in the brain (2). Some of the initial active immunotherapies targeted at brain amyloid plaques were associated with a high rate of unacceptable adverse events in clinical trials (e.g., meningoencephalitis (3).

Donanemab (LY3002813) is an immunoglobulin IgG1 antibody directed at an N-terminal pyroglutamate Aβ epitope that is present only in brain amyloid plaques. Donanemab was developed to remove existing amyloid plaques through microglial-mediated phagocytosis. Administration of the murine surrogate of donanemab in aged amyloid precursor protein transgenic mice resulted in dose-dependent plaque reduction without microhemorrhage liability (4). In the first-in-human single-dose and multiple-dose, placebo-controlled, dose-escalation Phase 1a study, donanemab 10-mg/kg was associated with 40–50% reductions in amyloid plaque deposits in amyloid-positive patients with mild cognitive impairment (MCI) due to AD or mild to moderate AD dementia (5). Overall, donanemab was generally well tolerated up to 10-mg/kg in this Phase 1a study. The most common treatment-emergent adverse events among 51 donanemab-treated participants were mild-to-moderate infusion reactions (6 of 37 patients with AD who had IV dosing) and asymptomatic cerebral microhemorrhage (2 out of 51 donanemab treated participants). No cases of vasogenic cerebral edema (ARIA-E) were reported. Approximately, 90% of participants developed anti-drug antibodies at 3 months following a single intravenous dose (5).

Based on the positive safety and pharmacodynamic (PD) findings from the Phase 1a study (5), a second Phase 1 study was initiated with donanemab in patients with MCI due to AD or mild to moderate AD. The overall goal of this Phase 1b study was to determine whether different dosing regimens (single-dose, dosing frequency, and chronic dosing for maximal PD effect) could mitigate immunogenicity, potential immune safety issues and produce sustained amyloid reduction. The primary objective was to assess the effect of donanemab on brain plaque load using florbetapir positron emission tomography (PET) imaging. The secondary objectives were to assess the safety, pharmacokinetics (PK), immunogenicity, and cognitive function effects of donanemab following single intravenous (IV) and multiple IV doses.

## Methods

### Study Design and Treatment

This Phase 1b study was conducted between December 22, 2015 and July 08, 2020 at 8 clinical research centers in the United States and Japan among patients with MCI due to AD or mild to moderate AD (6, 7). The study was a 3-part, patient- and investigator-blind, randomized within cohort, placebo-controlled, parallel-group, single- and multiple-dose study. Each of the 6 cohorts was designed to include approximately 6 (single dose) or 9 (multiple dose) patients treated with donanemab and 2 to 3 patients treated with placebo. Patients in Cohorts 1–3 were each administered a single, IV dose of donanemab (Cohort 1: 10-mg/kg, Cohort 2: 20-mg /kg, Cohort 3: 40-mg/kg) or placebo (Supplemental Figure 1). Follow-up was 72 weeks (Cohorts 1 and 2) or 24 weeks (Cohort 3). Patients in Cohort 4 were each administered multiple IV doses of donanemab (10-mg/kg) or placebo every 2 weeks (Q2W) for up to 24 weeks followed by a 48-week follow-up period to obtain amyloid clearance and safety data. Patients in Cohorts 5 and 6 were each administered multiple IV doses of donanemab (Cohort 5: 10-mg/kg; Cohort 6: 20-mg/kg) or placebo every 4 weeks (Q4W) for up to 72 weeks followed by a 12-week follow-up period.

**Figure 1:**
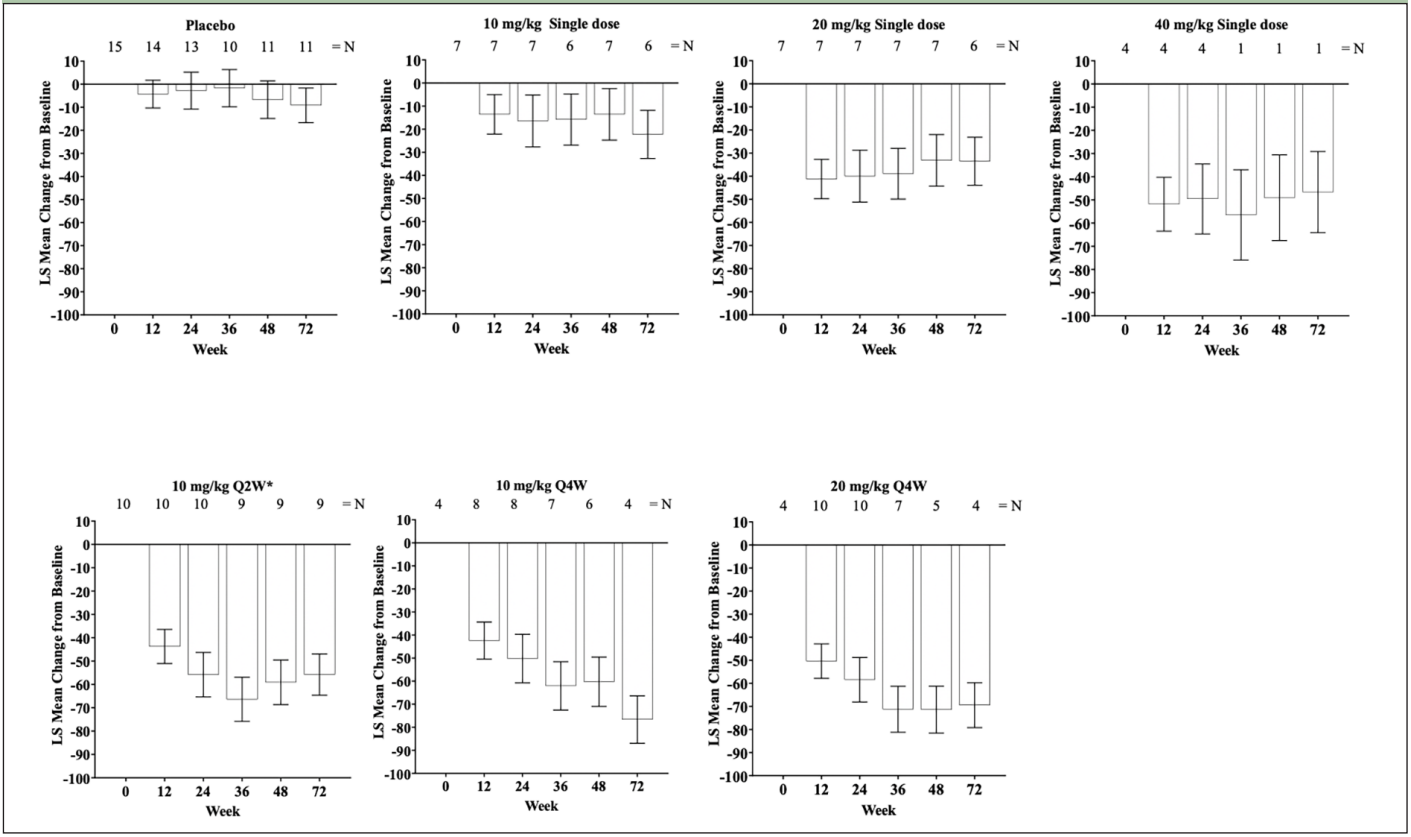
LS mean change of florbetapir PET scans from baseline (Centiloid units) through Week 72 following single and multiple dosing of IV donanemab

Error bars = SE; Treatment duration of 24 weeks; Abbreviations: IV = intravenous; LS mean = least squares mean; N = number of patients; PET = positron emission tomography; Q2W = every 2 weeks; Q4W = every 4 weeks; SE = standard error.

### Study Population

The study enrolled men or nonfertile women ≥50 years of age with evidence of memory impairment on the Free and Cued Selective Reminding Test with Immediate Recall (FCSRT-IR, picture version; <27 for free recall), a Mini-Mental State Examination (MMSE) score of 16 to 30, a Clinical Dementia Rating (CDR) of 0.5 to 2 and memory box score >0.5, and a florbetapir PET scan consistent with the presence of amyloid pathology (as determined using visual assessments and composite standardized uptake value ratio [SUVr] cut-points). The florbetapir F 18 interpretation method used for the eligibility decision included quantification as an adjunct to a visual assessment. The PET imaging core lab was responsible for performing both visual and quantitative analysis of the florbetapir F 18 images. All patients had gradual and progressive change in memory function reported by the patients themselves or informants over a period of more than 6 months. Patients with contraindication for magnetic resonance imaging (MRI), presence of more than four microhemorrhages on MRI, or history or evidence on MRI of macrohemorrhage were excluded. In addition to US patients, Japanese patients were included in this study to explore the safety and PK of donanemab in patients of Japanese decent.

### Study Evaluations

Florbetapir PET scans were performed at baseline and at 12, 24, 36, 48, and 72 weeks after starting treatment to estimate mean change in amyloid plaques. Calculation of SUVr with cerebellum reference region was as described previously (8). The latter SUVr values were converted to Centiloid units (9). Volumetric measurements were obtained from structural (3D T1-weighted) MR images acquired at screening and at 24, 48, and 72 weeks. Volume and atrophy were assessed in multiple brain regions including whole brain, lateral ventricles, and hippocampus.

Apolipoprotein E (APOE) genotyping was performed at baseline to determine genetic variants that may influence response to treatment.

Emergence of antibodies against donanemab was evaluated to asses the immunogenicity risk. Antidrug antibodies (ADAs) were detected using an affinity capture elution (ACE) Bridge assay validated at BioAgilytix Labs in Durham, North Carolina, USA. The ACE Bridge immunogenicity assay was developed based on published methods (10, 11, 12, 13). Serum for determination of ADAs was collected at screening/baseline and then at regular intervals throughout the study period.

Safety in the study was assessed at regular intervals with MRIs, electrocardiograms, safety laboratory tests (clinical chemistry, hematology, and urinalysis), physical/neurological examinations, and by monitoring the occurrence of adverse events, vital signs, and immunogenicity. In addition, the Columbia Suicide Severity Rating Scale (child version) and (if applicable) the Self-Harm Supplement Form were completed prior to dosing and at most study visits.

Cognition was assessed at screening or baseline for all patients using the CDR, the MMSE, the FCSRT-IR, the Alzheimer's Disease Assessment Scale Cognitive Subscale (ADAS-Cog-14), the Alzheimer's Disease Cooperative Study-Mild Cognitive Impairment-Activities of Daily Living, 24-item questionnaire (ADCS-MCI-ADL-24), and the Neuropsychological test battery (NTB). Additionally, these assessments were also performed at 24, 48, and 72 weeks after starting treatment or at the end of the study (eg, Week 24 for Cohort 3) or upon early discontinuation.

### Bioanalytical Methods

Serum and CSF samples were evaluated for donanemab using a validated enzyme-linked immunosorbent assay method at Covance Laboratories in Chantilly, Virginia, USA. The lower and upper limit of quantification for the serum assay was 200 ng/mL and 5000 ng/mL, respectively. During validation, the inter-assay accuracy (% relative error) ranged from −1.5%–7.0% and −2.9–5.3% and the inter-assay precision (% relative standard deviation) was 4.0–9.7% and 5.2–8.7%.

### Pharmacokinetic and Pharmacodynamic Analyses

Serum PK parameter estimates were calculated by standard noncompartmental methods using Phoenix WinNonlin Version 6.3 (Certara L.P., Raleigh, North Carolina, USA). Parameters estimated after IV administration included maximum observed drug concentration (Cmax), area under the concentration versus time curve (AUC) from time 0 to time infinity (AUC(0-∞)), and terminal half-life (t1/2). Mean plasma concentration versus time profiles and summary statistics of PK parameter estimates by treatment group were generated. To evaluate the potential effect of anti-donanemab antibodies on PK, observed trough donanemab concentrations were plotted by dose separately with time-matched anti-donanemab antibody results. A sample collection time window of 168–672 hours (1–4 weeks) and 168–1344 hours (1–8 weeks) from the most recent dose was used to identify trough concentrations for the Q2W and Q4W dosing regimens, respectively. Samples of CSF and serum were collected at baseline and approximately 72 hours following donanemab administration for the single dose cohorts or at baseline and approximately 72 hours following the dose administered at Week 24 for the multiple dose cohorts and assessed for donanemab concentration. These concentrations were compared to calculate a CSF:serum concentration ratio.

Composite SUVr from florbetapir scans were analyzed to estimate change (14) in amyloid burden. Furthermore, those SUVr values were converted to the Centiloid scale, a standardized methodology to quantify amyloid burden from PET scans (9).

### Statistical Analysis

This study intended to enroll approximately 72 patients, a sample size that is customary for studies evaluating safety, PK, and/or PD parameters. Based on prior clinical trials conducted by the sponsor, randomizing 6 patients to each donanemab dose was expected to provide approximately 90% power to detect 17% mean florbetapir SUVr reduction of a dose compared to placebo without multiple comparison adjustment.

The demographic variables, other baseline characteristics, and safety parameters were summarized using standard descriptive statistics. Safety analyses were conducted for all enrolled patients, whether or not they completed all protocol requirements.

PD analyses were conducted on the full analysis set, which included all data from all randomized patients receiving at least one dose of the investigational product according to the treatment the patients actually received. The PD measures included florbetapir PET scans in Centiloid units and were analyzed using a mixed model repeated measure (MMRM) with fixed effects of treatment doses, study visit, interaction between treatment and visit, baseline amyloid PET scan (Centiloid unit), and APOE-ε4 status (carrier /non-carrier) as covariate adjustment. An unstructured covariance matrix was used to model the within-subject variance-covariance errors.

Immunogenicity evaluation was based on antibody formation, that was summarized over time. Treatment-emergent ADAs (TE-ADAs) were defined as those with a titer 2-fold (1 dilution) greater than the minimum required dilution if no ADAs were detected at baseline or those with a 4-fold (2 dilutions) increase in titer compared to baseline if ADAs were detected at baseline. The minimum required dilution of the ADA assay was 1:5.

Cognitive outcomes (CDR, MMSE, FSCRT-IR, ADAS-Cog-11, ADCS-MCI-ADL-24, and NTB) were analyzed using a MMRM with baseline cognitive measures as a baseline covariate, fixed-effects of dose, visit, the dose-visit interaction, and appropriate covariance structures for model convergence. Statistical analyses were performed using SAS EG 9.4 software.

## Results

### Demographics and Baseline Characteristics

For patients receiving at least 1 dose of study drug, the demographic and baseline characteristics were generally balanced across the treatment groups (Table 1). A total of 61 patients (donanemab, n = 46; placebo, n = 15) participated in this study. Patients were male (n =27) and female (n = 34) with a mean age of 73.2 years (range: 54 to 90 years). Forty-three (70.5%) patients were non-Japanese and 18 (29.5%) patients were Japanese. At baseline, the mean MMSE total score was 21.1 (Standard Deviation [SD] = 4.04) and the mean florbetapir PET Centiloid units was 104.5 (SD = 32.77). Seventy-seven percent (47 of 61) of patients were APOE-ε4 carriers (11 homozygotes and 36 heterozygotes).

**Table 1:** Demographic and Baseline Characteristics

	Placebo*	Donanemab					
		10-mg/kg Single Dose	20-mg/kg Single Dose	40-mg/kg Single Dose	10-mg/kg Q2W	10-mg/kg Q4W	20-mg/kg Q4W
N	15	7	7	4	10	8	10
Age (years), mean (SD)	74.5 (10.1)	78.3 (8.4)	75.6 (6.4)	75.3 (6.7)	66.8 (8.6)	73.0 (7.8)	71.7 (9.2)
Female, n (%)	11 (73.3)	4 (57.1)	3 (42.9)	2 (50.0)	4 (40.0)	4 (50.0)	6 (60.0)
White, n (%)	9 (60.0)	5 (71.4)	6 (85.7)	1 (25.0)	7 (70.0)	5 (62.5)	7 (70.0)
Japanese, n (%)	5 (33.3)	2 (28.6)	1 (14.3)	2 (50.0)	3 (30.0)	3 (37.5)	2 (20.0)
Body mass index (kg/m^2^), mean (SD)	24.17 (3.46)	26.24 (6.52)	25.87 (3.81)	22.99 (2.55)	25.61 (3.36)	25.25 (3.62)	27.07 (3.99)
MMSE total score, mean (SD)	21.67 (4.81)	21.71 (4.23)	23.57 (4.50)	23.25 (3.86)	20.00 (3.40)	18.86 (2.41)	19.50 (2.93)
APOE-E4 status, n (%)							
ε4 carrier	10 (66.7)	5 (71.4)	5 (71.4)	4 (100)	8 (80.0)	6 (75.0)	9 (90.0)
Homozygotes (ε4/ε4)	3 (20.0)	1 (14.3)	2 (28.6)	0 (0)	2 (20.0)	1 (12.5)	2 (20.0)
Heterozygotes (ε2/ε4+ ε3/ε4)	7 (46.7)	4 (57.1)	3 (42.9)	4 (100)	6 (60.0)	5 (62.5)	7 (70.0)
ε4 noncarrier	5 (33.3)	2 (28.6)	2 (28.6)	0 (0)	2 (20.0)	2 (25.0)	1 (10.0)
Florbetapir PET Centiloid, mean (SD)	104.42 (32.29)	101.01 (39.16)	99.70 (27.48)	93.68 (40.58)	108.70 (20.89)	103.52 (50.03)	111.08 (30.54)


*Data from the single dose, Q2W, and Q4W placebo arms were pooled; Abbreviations: APOE = apolipoprotein E; MMSE = Mini-Mental State Examination; N = number of patients; n = number of patients in a subgroup; PET = positron emission tomography; Q2W = every 2 weeks; Q4W = every 4 weeks; SD = standard deviation.

### Disposition

Among 276 patients screened, 61 patients satisfied entry criteria and were enrolled into the study (7, 7, and 4 patients were randomized to the 10-mg/kg, 20-mg/ kg, and 40-mg/kg single dose cohorts respectively; 10 patients were randomized to the 10-mg/kg Q2W for 24 weeks cohort and 8 and 10 patients were randomized to the 10-mg/kg Q4W and 20-mg/kg Q4W cohorts respectively). For simplicity, all patients receiving placebo were pooled into one group. Main reasons for screen failure were not meeting threshold criteria for amyloid PET (40 of 154 patients; 26.0%), cognition (MMSE/FCSRT-IR; 33 of 154 patients; 21.4%), and microhemorrhage greater than 4 on MRI (16 of 154 patients; 10.4%). Of the 61 patients who received at least 1 dose of study treatment, 46 (75.4%) patients completed the study (Supplemental Figure 2). Fifteen patients did not complete the study, which included 6 due to investigator decision (3 in the 10-mg/kg Q4W cohort and 3 in the 20-mg/kg Q4W cohort); 5 due to the patient's withdrawal of consent (1 in the 10-mg/kg single dose cohort, 1 in the 10-mg/kg Q2W cohort, 1 in the 10-mg/kg Q4W cohort, 1 in the 20-mg/kg Q4W cohort, and 1 placebo); 3 patients discontinued due to adverse events (ARIA-E [20-mg/ kg Q4W cohort], hypertensive crisis [20-mg/kg Q4W cohort] and myocardial infarction, considered a serious adverse event, resulting in death [placebo Q4W cohort]); and 1 patient was lost to follow-up (20-mg/kg single dose cohort).

**Figure 2:**
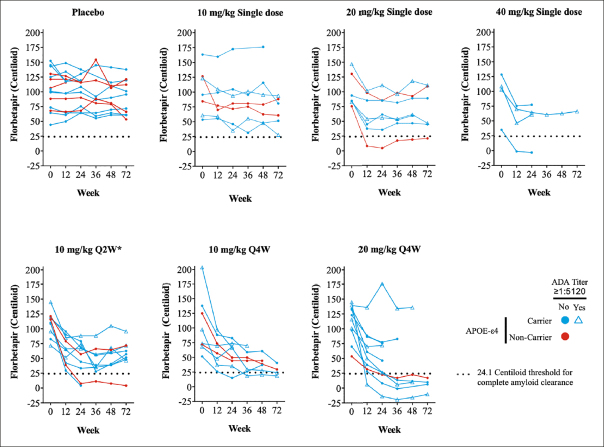
Cerebral amyloid over time as measured by quantitative amyloid PET imaging (florbetapir SUVr). Absolute Centiloid value as calculated from SUVr

*Treatment duration of 24 weeks; Notes: Color indicates APOE-ε4 status and symbol indicates ADA titer of ≥1:5120. The black dashed horizontal line indicates threshold Centiloid value for being amyloid positive; Abbreviations: APOE = apolipoprotein E; LY = LY3002813 (donanemab); PET = positron emission tomography; Q2W = every 2 weeks; Q4W = every 4 weeks; SUVr = standardized uptake value ratio.

### Florbetapir Positron Emission Tomography - Centiloid Scale and Standardized Uptake Value Ratio

Single and multiple doses of donanemab showed a consistent reduction from baseline in cerebral amyloid (Centiloid units) observed by PET from Week 12 through Week 72 (Figure 1). At Week 24, amyloid PET least squares mean Centiloid changes from baseline for single donanemab doses were: −16.5 (standard error [SE] = 11.22) 10-mg/kg IV; −40.0 (SE = 11.23) 20-mg/kg IV; and −49.6 (SE = 15.10) 40-mg/kg IV. In contrast, in the placebo group there was no significant reduction in florbetapir PET at 72 weeks (90.9 Centiloids at 72 weeks compared to 104.4 Centiloids at baseline). Corresponding Centiloid changes for multiple doses at Week 24 included: −55.8 (SE = 9.51) 10-mg/kg Q2W; −50.2 (SE = 10.54) 10-mg/kg Q4W; and −58.4 (SE = 9.66) 20-mg/kg Q4W. Patients in the 20 mg/kg Q4W cohort tended to achieve greater plaque reduction earlier in the study than patients in either of the 10 mg/kg multiple dose cohorts (Figures 1 and 2). After dosing, a sustained reduction of brain amyloid level without significant reaccumulation for up to 72 weeks was observed across all single- and multiple-dose cohorts.

The change in absolute Centiloid value did not appear to be influenced by APOE-ε4 status with no clear association between presence of the APOE-ε4 allele and florbetapir PET response (Figure 2). TE-ADAs (see below) also appeared not to impact the reduction in amyloid as some participants with high TE-ADA titers (≥1:5120) still had a reduction in amyloid in this study (Figure 2).

Overall, 2 participants in single-dose cohorts (1 in 20-mg /kg and 1 in 40-mg /kg) and 9 participants in the multiple-dose cohorts (2 in 10mg/kg Q2W; 2 in 10-mg /kg Q4W; and 5 in 20-mg /kg Q4W) achieved complete amyloid clearance status based on a threshold 24.1 Centiloid value. Most participants achieving amyloid clearance starting at 12 or 24 weeks remained amyloid negative for the duration of their florbetapir PET measurements.

Reduction in cerebral amyloid (Centiloid units) and SUVr changes from baseline were visually comparable between non-Japanese and Japanese patients (Supplemental Figure 3).

**Figure 3:**
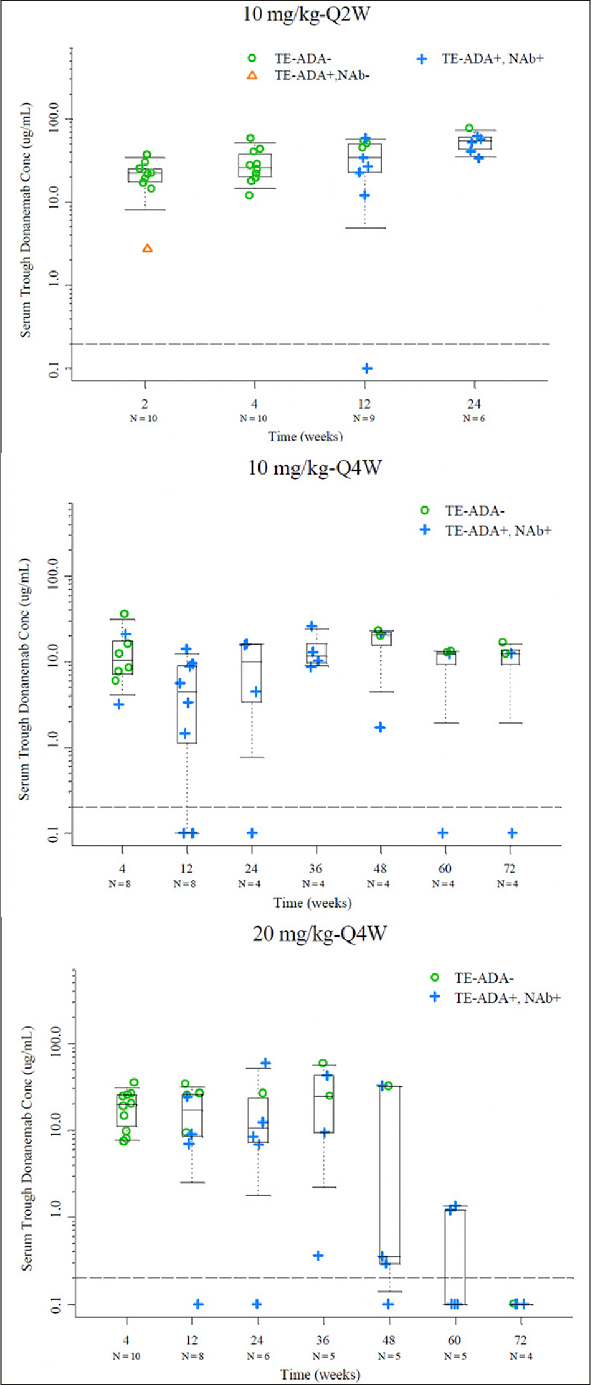
Serum trough concentrations with available time matched PK and TE ADA evaluable data in the 10 mg/kg Q2W, 10 mg/kg Q4W, and 20 mg/kg Q4W cohorts

Note: Dashed line represents BQL (0.2 µg/mL); Abbreviations: BQL = below the limit of quantification; N = number of patients; NAb = neutralizing antidrug antibody; PK = pharmacokinetics; Q2W = every 2 weeks; Q4W = every 4 weeks; TE-ADA = treatment emergent antidrug antibody.

### Single- and Multiple-Dose Serum and Cerebrospinal Fluid PK

Dose proportional increases were observed in both Cmax and exposure (AUC) following single and multiple doses. Single doses of 10, 20, and 40 mg/kg had measurable donanemab concentration for at least 56 days post-dose with elimination t1/2 of approximately 10 days. Multiple doses resulted in either no (10 mg/kg Q4W) or very limited exposure accumulation (10 mg/ kg Q2W; 20 mg/kg Q4W). PK parameters for single and multiple dose cohorts are summarized in Supplemental Tables 1 and 2, respectively. Single dose PK characteristics were similar between Japanese and non-Japanese participants, albeit based on small sample size (5 patients who are Japanese out of 18 patients given donanemab). Quantifiable concentrations were detected in CSF samples collected from patients treated with single and multiple donanemab doses with CSF to serum concentration ratio of approximately 0.2% across all patients and dose levels.

**Table 2:** Treatment-Emergent Adverse Events

	Placebo*	Donanemab					
		10-mg/kg Single Dose	20-mg/kg Single Dose	40-mg/kg Single Dose	10-mg/kg Q2W	10-mg/kg Q4W	20-mg/kg Q4W
N	15	7	7	4	10	8	10
Patients with ≥1 SAE, n (%)	3 (20.0)	0 (0)	0 (0)	0 (0)	0 (0)	1 (12.5)	2 (20.0) †
Patients with ≥1 TEAE, n (%)	11 (73.3)	7 (100)	6 (85.7)	4 (100)	10 (100)	8 (100)	9 (90.0)
Total TEAEs per Cohort, n	37	15	28	6	50	53	34
Common TEAEs, Regardless of Causality; n [Number of Patients with Event] (%)							
Vasogenic cerebral edema (ARIA-E)	0 (0)	0 (0)	2 [2] (28.6)	2 [2] (50.0)	2 [2] (20.0)	3 [3] (37.5)	3 [3] (30.0)
Cerebral microhemorrhage	1 [1] (6.7)	2 [1] (14.3)	3 [1] (14.3)	0 (0)	2 [2] (20.0)	0 (0)	3 [2] (20.0)
Headache	1 [1] (6.7)	4 [1] (14.3)	2 [2] (28.6)	0 (0)	1 [1] (10.0)	0 (0)	3 [1] (10.0)
Upper repiratory tract infection	3 [3] (20.0)	0 (0)	0 (0)	0 (0)	0 (0)	2 [2] (25.0)	1 [1] (10.0)
Vomiting	0 (0)	0 (0)	0 (0)	0 (0)	2 [1] (10.0)	1 [1] (12.5)	2 [1] (10.0)
Back pain	0 (0)	1 [1] (14.3)	1 [1] (14.3)	0 (0)	0 (0)	0 (0)	1 [1] (10.0)
Fatigue	1 [1] (6.7)	0 (0)	0 (0)	0 (0)	2 [2] (20.0)	0 (0)	0 (0)
Oedema peripheral	0 (0)	0 (0)	0 (0)	0 (0)	1 [1] (10.0)	1 [1] (12.5)	1 [1] (10.0)
Post lumbar puncture syndrome	1 [1] (6.7)	1 [1] (14.3)	0 (0)	1 [1] (25.0)	0 (0)	0 (0)	0 (0)
Viral upper respiratory tract infection	0 (0)	0 (0)	0 (0)	1 [1] (25.0)	1 [1] (10.0)	1 [1] (12.5)	0 (0)
Basal cell carcinoma	0 (0)	0 (0)	2 [1] (14.3)	0 (0)	0 (0)	0 (0)	1 [1] (10.0)
Fall	0 (0)	0 (0)	0 (0)	0 (0)	2 [1] (10.0)	1 [1] (12.5)	0 (0)
Agitation	0 (0)	0 (0)	0 (0)	0 (0)	1 [1] (10.0)	1 [1] (12.5)	0 (0)
Anaemia	0 (0)	0 (0)	0 (0)	0 (0)	1 [1] (10.0)	1 [1] (12.5)	0 (0)
Anxiety	0 (0)	0 (0)	0 (0)	1 [1] (25.0)	1 [1] (10.0)	0 (0)	0 (0)
Atrophic vulvovaginitis	2 [2] (13.3)	0 (0)	0 (0)	0 (0)	0 (0)	0 (0)	0 (0)
Contusion	0 (0)	0 (0)	0 (0)	0 (0)	1 [1] (10.0)	1 [1] (12.5)	0 (0)
Cough	0 (0)	0 (0)	0 (0)	0 (0)	1 [1] (10.0)	1 [1] (12.5)	0 (0)
Dizziness	0 (0)	0 (0)	1 [1] (14.3)	0 (0)	0 (0)	1 [1] (12.5)	0 (0)
Ecchymosis	0 (0)	0 (0)	0 (0)	0 (0)	1 [1] (10.0)	1 [1] (12.5)	0 (0)
Irritability	0 (0)	1 [1] (14.3)	0 (0)	0 (0)	0 (0)	1 [1] (12.5)	0 (0)
Muscle spasms	0 (0)	0 (0)	0 (0)	0 (0)	0 (0)	1 [1] (12.5)	1 [1] (10.0)
Procedural anxiety	0 (0)	0 (0)	0 (0)	0 (0)	1 [1] (10.0)	0 (0)	1 [1] (10.0)
Rash	2 [2] (13.3)	0 (0)	0 (0)	0 (0)	0 (0)	0 (0)	0 (0)
Skin abrasion	0 (0)	0 (0)	0 (0)	0 (0)	1 [1] (10.0)	1 [1] (12.5)	0 (0)
Somnolence	1 [1] (6.7)	0 (0)	0 (0)	1 [1] (25.0)	0 (0)	0 (0)	0 (0)
Superficial siderosis of CNS	0 (0)	0 (0)	0 (0)	0 (0)	0 (0)	1 [1] (12.5)	1 [1] (10.0)
Urinary tract infection	1 [1] (6.7)	0 (0)	1 [1] (14.3)	0 (0)	0 (0)	0 (0)	1 [1] (10.0)
Anger	0 (0)	0 (0)	0 (0)	0 (0)	0 (0)	2 [2] (25.0)	0 (0)
Renal injury	0 (0)	0 (0)	0 (0)	0 (0)	0 (0)	3 [1] (12.5)	0 (0)
Bradycardia	0 (0)	0 (0)	0 (0)	0 (0)	0 (0)	0 (0)	2 [1] (10.0)
Delusional perception	0 (0)	0 (0)	2 [1] (14.3)	0 (0)	0 (0)	0 (0)	0 (0)
Dizziness exertional	0 (0)	0 (0)	2 [1] (14.3)	0 (0)	0 (0)	0 (0)	0 (0)
Epistaxis	0 (0)	0 (0)	0 (0)	0 (0)	2 [1] (10.0)	0 (0)	0 (0)
Hyponatraemia	2 [1] (6.7)	0 (0)	0 (0)	0 (0)	0 (0)	0 (0)	0 (0)
Infusion site oedema	0 (0)	0 (0)	0 (0)	0 (0)	0 (0)	2 [1] (12.5)	0 (0)
Infusion site reaction	0 (0)	0 (0)	0 (0)	0 (0)	2 [1] (10.0)	0 (0)	0 (0)
Procedural complication	0 (0)	0 (0)	0 (0)	0 (0)	0 (0)	2 [1] (12.5)	0 (0)
Pruritus	2 [1] (6.7)	0 (0)	0 (0)	0 (0)	0 (0)	0 (0)	0 (0)
Number of Severe TEAEs	2	1	1	0	0	0	3
Discontinuation Due to TEAE, n (%)	0 (0)	0 (0)	0 (0)	0 (0)	0 (0)	0 (0)	2 (20.0)
Patients with ≥1 Study Drug-Related TEAE, n (%)	4 (26.7)	2 (28.6)	3 (42.9)	2 (50.0)	6 (60.0)	3 (37.5)	4 (40.0)
Common Study-Drug Related TEAEs, n [Number of Patients with Event] (%)							
Vasogenic cerebral edema (ARIA-E)	0 (0)	0 (0)	2 [2] (28.6)	2 [2] (50.0)	2 [2] (20.0)	3 [3] (37.5)	3 [3] (30.0)
Cerebral microhemorrhage	0 (0)	2 [1] (14.3)	2 [1] (14.3)	0 (0)	2 [2] (20.0)	0 (0)	3 [2] (20.0)
Vomiting	0 (0)	0 (0)	0 (0)	0 (0)	1 [1] (10.0)	0 (0)	2 [1] (10.0)
Dizziness	0 (0)	0 (0)	1 [1] (14.3)	0 (0)	0 (0)	1 [1] (12.5)	0 (0)
Superficial siderosis of CNS	0 (0)	0 (0)	0 (0)	0 (0)	0 (0)	1 [1] (12.5)	1 [1] (10.0)

*Data from the single dose Q2W and Q4W placebo arms were pooled; †One patient reported 2 SAEs; Abbreviations: ARIA = amyloid-related imaging abnormalities; CNS = central nervous system; E = vasogenic cerebral edema; H = cerebral microhemorrhage; N = number of patients; n = number of patients in a group; Q2W = every 2 weeks; Q4W = every 4 weeks; SAE = serious adverse event; TEAE = treatment emergent adverse event.

### Treatment-emergent Antidrug Antibodies and Effect on Donanemab Serum Concentration

Postbaseline, 46 donanemab-treated participants were evaluable for TE-ADAs. Except for 1 patient in the 10-mg/kg single-dose cohort, all other 45 patients randomized to donanemab developed TE-ADAs. All 6 treatment groups randomized to single or multiple IV administration of donanemab exhibited distinctly higher TE-ADA titers relative to the placebo group. No relationship between dose and TE-ADA was identified in this study. The overall incidence of TE-ADA and titer dynamics were similar for each dose group. The majority of participants exhibited TE-ADAs 3 months after the first dose of donanemab, which returned to or towards baseline after discontinuation of treatment. All 45 donanemab-treated TE-ADA-positive participants were also positive for neutralizing antibody (Nab) to donanemab. Maximum titers for TE-ADA+ participants ranged from 1:10-1:327680 with a median maximum titer of 1:2560. A total of 17 out of 46 of AD patients exposed to donanemab developed high titers (≥1:5120).

To evaluate the potential effect of the kinetics (onset and duration) of TE-ADA on donanemab PK after multiple dosing in the 10 mg/kg Q2W, 10 mg/kg Q4W and 20 mg/kg Q4W cohorts, the observed trough drug concentrations were plotted by dose with ADA results (time-matched with PK) for each visit. Based on graphical analyses, overall there did not appear to be a significant effect of TE-ADAs on the PK of donanemab despite the high incidence of TE-ADAs. Observed trough concentrations among TE-ADA+, NAb+ samples (N=59) appeared similar to those that were TE- ADA-(N=54) across all multiple dose groups (one sample was TE-ADA+, NAb−). Exceptions were observed following 20 mg/kg Q4W beyond Week 48 (Figure 3) where mean trough donanemab concentrations of TE-ADA+, NAb+ samples appeared lower compared with those earlier than Week 48. However, these observations are based on a small number of trough samples, namely Week 48 (5 samples), Week 60 (5 samples), and Week 72 (4 samples). Specific individual participants associated with these lower trough samples were identified to graphically evaluate any effect of titer value on low trough donanemab concentrations. Out of these participants with lower than previous trough samples, there were 2 participants with concentrations below the limit of quantification and low titers, as well as 2 participants with low but quantifiable concentrations and high titers (selected data shown in Supplemental Figure 4).

**Figure 4:**
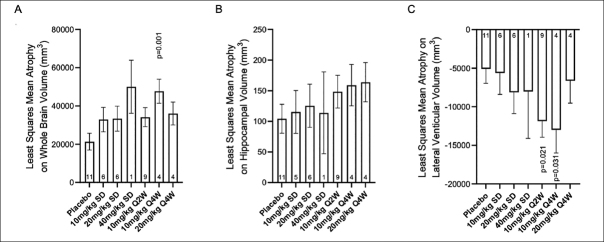
Least squares mean atrophy on A) whole brain volume, B) average hippocampal volume, and C) lateral ventricle volume (mm3) per study intervention group at 72 weeks

Abbreviations: Q2W = every 2 weeks; Q4W = every 4 weeks; p-values are versus placebo

### Safety

A total of 7 serious adverse events among 6 patients were reported. Of these, 1 patient (randomized to placebo) discontinued from the study because of a SAE of death due to myocardial infarction (considered not drug-related by the investigator). One of the SAEs, intermittently symptomatic ARIA-E was considered drug-related (20-mg/kg Q4W cohort). The remaining 5 SAEs were considered not drug-related by the investigator.

A total of 223 treatment-emergent adverse events (TEAEs) across all cohorts were reported in this study, regardless of causality (Table 2). Of 61 patients, 55 patients (90.2%) reported least 1 TEAE (generally mild to moderate in severity) and 24 (39.3%) reported at least 1 study drug-related TEAE. The most common TEAE of ARIA-E was experienced by 12 out of 46 donanemab-treated patients with AD and occurred in all donanemabdosing cohorts except the 10-mg/kg single-dose cohort. The most common study drug-related TEAEs after a single dose of study drug were ARIA-E (n = 4) and cerebral microhemorrhage (n = 4). The most common study drug-related TEAEs in the Q2W- and Q4W-dose cohorts were ARIA-E (n = 2 and n = 6, respectively) and cerebral microhemorrhage (n = 2 and n = 3, respectively).

One infusion-related reaction was reported in 1 patient in the 10-mg/kg Q2W cohort. An additional event of hypertensive crisis had timing consistent with an infusion-related reaction. Three patients discontinued the study prematurely due to an AE: fatal myocardial infarction (placebo Q4W cohort), mild hypertensive crisis (20-mg/kg Q4W cohort), and mild ARIA-E (20-mg/kg Q4W cohort). The patients with hypertensive crisis and ARIA-E both recovered after approximately 20 mins and 8 weeks, respectively. There were no clinically significant changes in other safety assessments, including vital signs, safety laboratories, electrocardiograms, and neurological examinations. Overall, all safety analyses showed no clinically relevant differences between non-Japanese and Japanese patients.

### ARIA

Overall, ARIA-E events occurred in 12 of the 46 donanemab-treated patients of whom 2 were symptomatic with mild to moderate symptoms (headache, confusion, hyper-somnolence, and nausea) (Table 2). All patients with ARIA-E were discontinued from study drug as per protocol. All ARIA-E events (including symptoms) resolved following dose discontinuation. All events were considered drug-related.

There were 10 events of cerebral microhemorrhage among 6 of the 46 donanemab-treated patients (Table 2). The majority of cerebral microhemorrhage events (9 of 10) were considered drug-related. Superficial siderosis was reported for 1 patient in the 10-mg/kg Q4W cohort and 1 patient in the 20-mg/kg Q4W cohort. Macrohemorrhage was not observed.

### Volumetric Magnetic Resonance Imaging (vMRI)

Overall, administration of donanemab did not result in consistent significant reductions in whole brain volume or hippocampal brain volume nor were there consistent significant increases in lateral ventricular volume when compared to placebo (Figure 4 and Supplemental Figure 5). The changes in whole brain, hippocampal, and ventricular volume were generally numerically greater at 72 weeks with donanemab treatment compared to placebo. However, there was no dose response in the changes, and there were no significant changes in most donanemab treatment cohorts.

### Cognition and function

Across all dose groups, there were no significant changes from baseline in any of the cognitive measures with donanemab treatment (data not shown).

## Discussion

This Phase 1b study was a randomized, placebo-controlled, single- and multiple-dose study in patients with MCI due to AD or mild to moderate AD (amyloid detected by a positive florbetapir scan). PD, PK, immunogenicity, safety, and tolerability of single and multiple IV doses of donanemab were assessed. The main findings in this study were that:

1) single and multiple doses of donanemab up to 40 mg and 20-mg/kg Q4W, respectively, reduced amyloid plaque deposits in patients with AD; 5 out of 10 patients in the 20 mg/kg Q4W cohort attained complete amyloid clearance within 36 weeks
2) the observed amyloid plaque lowering by donanemab was rapid, robust, and sustained
3) nearly all donanemab-treated patients developed anti-drug antibodies, however, there was no overall significant effect of the antibodies on the PK of donanemab for the duration of the study, given the observed linear PK
4) donanemab was generally well tolerated with manageable ARIA-E events that resolved completely upon treatment discontinuation.

A reduction in cerebral amyloid plaque has also been reported with other anti-amyloid monoclonal antibodies, like gantenerumab, lecanemab, and aducanumab (15, 16, 17, 18). The findings of a rapid and dose-dependent reduction in cerebral amyloid plaque after donanemab treatment extend those of a previous ascending dose donanemab study (5), which demonstrated a similar reduction in cerebral amyloid at 10 mg/kg (the highest dose administered in that study). A novel finding in this study is that a significant reduction in cerebral amyloid plaque was observed, even after single doses of donanemab, and the reduction was sustained up to 72 weeks after the single dose. Importantly, the rate of the observed amyloid plaque lowering was rapid, with a greater than 50 Centiloid reduction observed after 24-weeks of multiple-dose donanemab treatment. Furthermore, complete amyloid clearance, as measured by florbetapir PET, was observed for 5 of 10 patients (50.0%) treated with 20-mg/ kg Q4W donanemab. This result was sustained through 18 months.

Notably, these robust effects of donanemab on cerebral amyloid were observed in the background of a high incidence of TE-ADAs. Although nearly all donanemab-treated patients developed anti-drug antibodies, there did not appear to be a clinically meaningful effect of the antibodies on the PK of donanemab. However, further analysis are planned where these and other longitudinal data will be analysed via population PK analyses with immunogenicity evaluated as a potential covariate. Despite the background of high TE-ADAs, the PK after single and multiple doses of donanemab were linear from 10- to 40-mg/kg. This result extends the dose range from the earlier Phase 1a study, where the PK of donanemab appeared to be non-linear in nature (5). The reason for this nonlinearity was unclear, and it was speculated that it might be attributed to either donanemab target-mediated disposition and/or anti-drug antibodies impacting PK (5). In this study, the high incidence of anti-drug antibodies was not associated with a high incidence of infusion-related reactions or hypersensitivity reactions (including anaphylaxis).

Donanemab was generally well tolerated with ARIA-E reported as the most common adverse event, which completely resolved upon treatment discontinuation. The incidence of ARIA-E (12 of 46 donanemab-treated patients; 26.1%) was within the range of rates of ARIA-E observed with other amyloid lowering antibodies (19). Several studies with amyloid-lowering therapies have shown a reduction in brain volume and/or an increase in ventricular volume with treatment (20, 21, 22, 23). There were no consistent significant changes in vMRI measurements in this study. However, vMRI was an exploratory endpoint in the study and the sample size was small, thus the effect of donanemab on brain volume will need to be more fully addressed in larger clinical studies.

There was no statistically significant effect of donanemab on cognition and function at any dose level or dosing regimen, although this is not unexpected given the small sample size and range of disease stages from MCI to moderate AD dementia enrolled in this study. In contrast, a larger, clinically and pathologically more homogenous Phase 2 trial TRAILBLAZER-ALZ (NCT03367403) met the prespecified primary endpoint of change from baseline to 76 weeks in the Integrated Alzheimer's Disease Rating Scale with a statistically significant slowing of decline by 32% relative to placebo. Donanemab-treated patients also showed consistent improvements in all prespecified secondary endpoints measuring cognition and function compared to placebo but did not reach nominal statistical significance on every secondary endpoint (24).

## Conclusion

Single and multiple doses of donanemab demonstrated a rapid and robust reduction in brain amyloid plaque. Single and multiple doses of donanemab yielded sustained amyloid plaque reduction without evidence of significant reaccumulation when measured at 72 weeks. The presence of ADAs were consistent with previous studies, and events of ARIA were manageable. These findings support donanemab dosing up to 1400 mg (approximately 20 mg/kg) Q4W in the TRAILBLAZER-ALZ phase 2 study (NCT03367403), TRAILBLAZER-EXT extension study (NCT04437511), the TRAILBLAZER-ALZ 2 Phase 3 study (NCT04640077) and the planned TRAILBLAZER-ALZ 3 study.

## Electronic supplementary material

Supplementary material, approximately 922 KB.

*Funding:* This work was supported by Eli Lilly and Company. The sponsors of the study were involved in the design and conduct of the study as well as the collection, analysis, and interpretation of data; in the preparation of the manuscript; and in the review or approval of the manuscript.

*Ethical Standards:* The study protocol was reviewed and approved by the ethics review board for each of the study sites. The studies were conducted according to Good Clinical Practice, consensus ethics principles derived from international ethics guidelines, including the Declaration of Helsinki and Council for International Organizations of Medical Sciences International Ethical Guidelines, ICH GCP Guideline [E6], and applicable laws and regulations. Patients and/or patients' legally acceptable representatives provided written informed consent before undergoing study procedures.

## References

[1] Karran E, Mercken M, De Strooper B (2011). The amyloid cascade hypothesis for Alzheimer's disease: an appraisal for the development of therapeutics. Nat Rev Drug Discov.

[2] Cummings J, Lee G, Ritter A, Sabbagh M, Zhong K (2019). Alzheimer's disease drug development pipeline: 2019. Alzheimers Dement (N Y).

[3] Gilman S, Koller M, Black RS (2005). Clinical effects of Abeta immunization (AN1792) in patients with AD in an interrupted trial. Neurology.

[4] Demattos RB, Lu J, Tang Y (2012). A plaque-specific antibody clears existing beta-amyloid plaques in Alzheimer's disease mice. Neuron.

[5] Lowe SL, Willis BA, Hawdon A (2021). Donanemab (LY3002813) dose-escalation study in Alzheimer's disease. Alzheimers Dement (N Y).

[6] Albert MS, DeKosky ST, Dickson D (2011). The diagnosis of mild cognitive impairment due to Alzheimer's disease: recommendations from the National Institute on Aging-Alzheimer's Association workgroups on diagnostic guidelines for Alzheimer's disease. Alzheimers Dement.

[7] McKhann GM (2011). Changing concepts of Alzheimer disease. JAMA.

[8] Barthel H, Gertz HJ, Dresel S (2011). Cerebral amyloid-beta PET with florbetaben (18F) in patients with Alzheimer's disease and healthy controls: a multicentre phase 2 diagnostic study. Lancet Neurol.

[9] Navitsky M, Joshi AD, Kennedy I (2018). Standardization of amyloid quantitation with florbetapir standardized uptake value ratios to the Centiloid scale. Alzheimers Dement.

[10] Bourdage JS, Cook CA, Farrington DL, Chain JS, Konrad RJ (2007). An Affinity Capture Elution (ACE) assay for detection of anti-drug antibody to monoclonal antibody therapeutics in the presence of high levels of drug. J Immunol Methods.

[11] Butterfield AM, Chain JS, Ackermann BL, Konrad RJ (2010). Comparison of assay formats for drug-tolerant immunogenicity testing. Bioanalysis.

[12] Chen YQ, Pottanat TG, Carter QL (2016). Affinity capture elution bridging assay: A novel immunoassay format for detection of anti-therapeutic protein antibodies. J Immunol Methods.

[13] Sloan JH, Conway RG, Pottanat TG (2016). An innovative and highly drug-tolerant approach for detecting neutralizing antibodies directed to therapeutic antibodies. Bioanalysis.

[14] Clark CM, Schneider JA, Bedell BJ (2011). Use of florbetapir-PET for imaging beta-amyloid pathology. JAMA.

[15] Bohrmann B, Baumann K, Benz J (2012). Gantenerumab: a novel human anti-Abeta antibody demonstrates sustained cerebral amyloid-beta binding and elicits cell-mediated removal of human amyloid-beta. J Alzheimers Dis.

[16] Ostrowitzki S, Deptula D, Thurfjell L (2012). Mechanism of amyloid removal in patients with Alzheimer disease treated with gantenerumab. Arch Neurol.

[17] Sevigny J, Chiao P, Bussiere T (2016). The antibody aducanumab reduces Abeta plaques in Alzheimer's disease. Nature.

[18] Swanson CJ, Zhang Y, Dhadda S (2018). DT-01-07: Treatment of early AD subjects with BAN2401, an anti-Aβ protofibrial monoclonal antibody, significantly clears amyloid plaque and reduces clinical decline. Alzheimer's & Dementia.

[19] Tolar M, Abushakra S, Hey JA, Porsteinsson A, Sabbagh M (2020). Aducanumab, gantenerumab, BAN2401, and ALZ-801-the first wave of amyloid-targeting drugs for Alzheimer's disease with potential for near term approval. Alzheimers Res Ther.

[20] Sur C, Kost J, Scott D (2020). BACE inhibition causes rapid, regional, and nonprogressive volume reduction in Alzheimer's disease brain. Brain.

[21] Zimmer JA, Shcherbinin S, Devous MD (2021). Lanabecestat: Neuroimaging results in early symptomatic Alzheimer's disease. Alzheimers Dement (N Y).

[22] Novak G, Fox N, Clegg S (2016). Changes in Brain Volume with Bapineuzumab in Mild to Moderate Alzheimer's Disease. J Alzheimers Dis.

[23] Swanson CJ, Zhang Y, Dhadda S (2021). A randomized, double-blind, phase 2b proof-of-concept clinical trial in early Alzheimer's disease with lecanemab, an anti-Abeta protofibril antibody. Alzheimers Res Ther.

[24] Mintun MA, Lo AC, Duggan Evans C (2021 Mar 13). Donanemab in Early Alzheimer's Disease. N Engl J Med.

