# Supplementary material for: Donanemab (LY3002813) Phase 1b Study in Alzheimer's Disease: Rapid and Sustained Reduction of Brain Amyloid Measured by Florbetapir F18 Imaging
Source: J Prev Alzheimers Dis. 2021 Sep 21;8(4):414–24. doi: 10.14283/jpad.2021.56 (PMC12280776; doi:10.14283/jpad.2021.56)
Supplement: Supplementary file 1 — Supplementary material, approximately 922 KB. [file mmc1.docx]

**
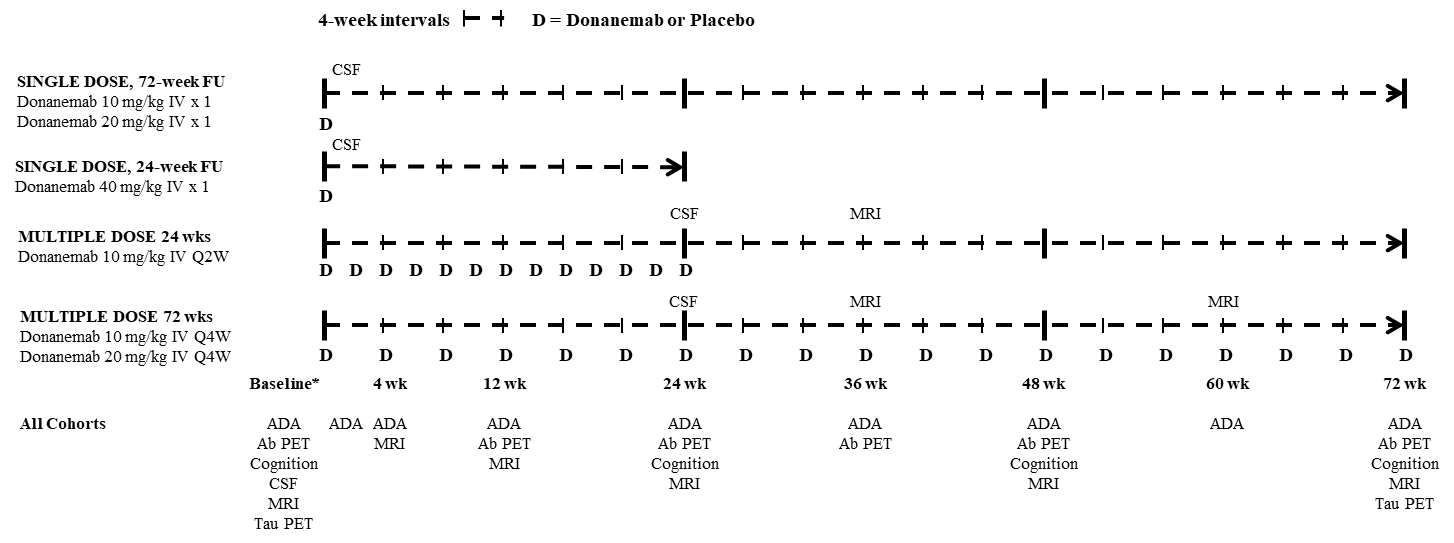
**

**Supplemental Figure 1.** Study Design - Overview of dosing regime and schedule of activities.

* Baseline = any visit before dosing

ADA = antidrug antibodies; Ab = Amyloid ß (florbetapir); CSF = cerebrospinal fluid; FU = follow up; IV = intravenous; MRI = magnetic resonance imaging; PET = positron emission tomography; Tau = 18-AV-1451 (flortaucipir); wk = week.

**
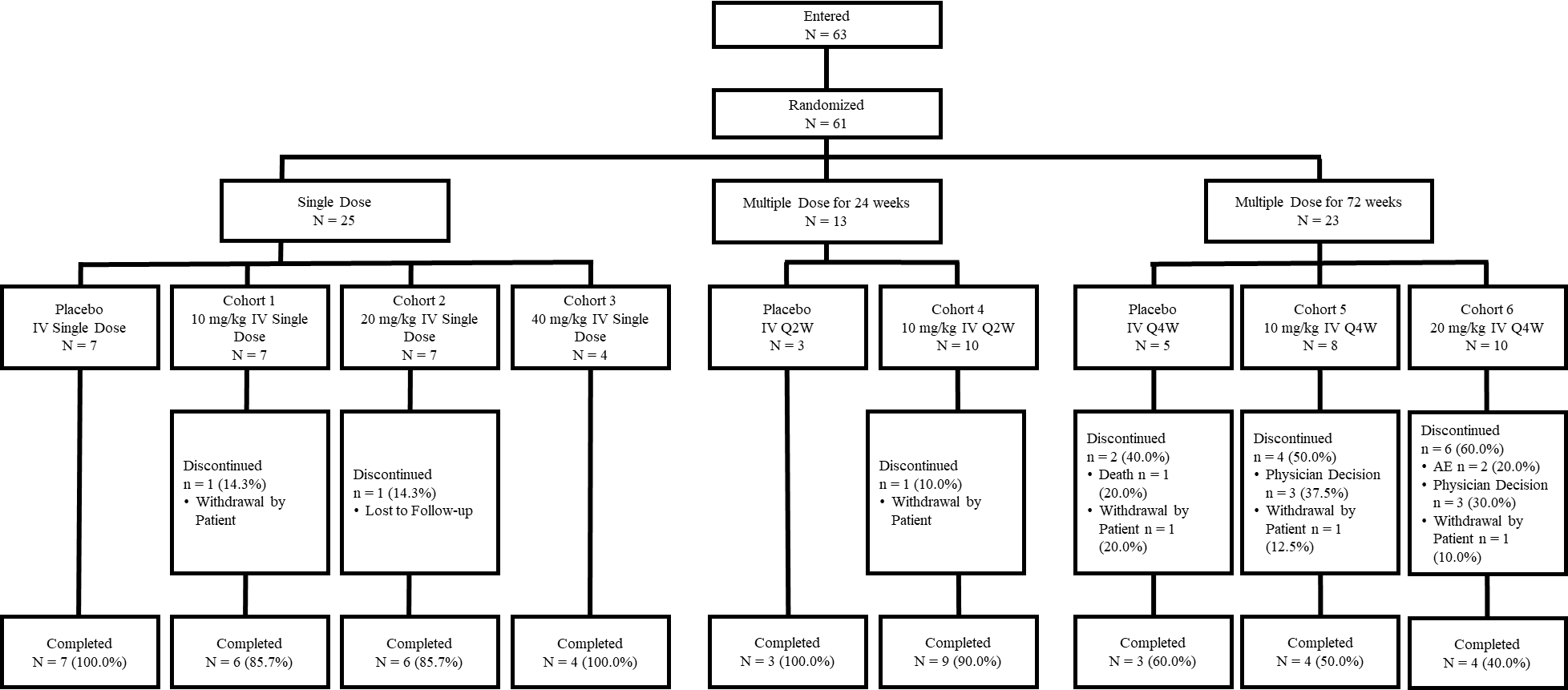
Supplemental Figure 2. Disposition of study participants**

Abbreviations: AE = adverse event; IV = intravenous; N = number of participants; n = number of patients in a subgroup; Q2W = every 2 weeks; Q4W = every 4 weeks.

**
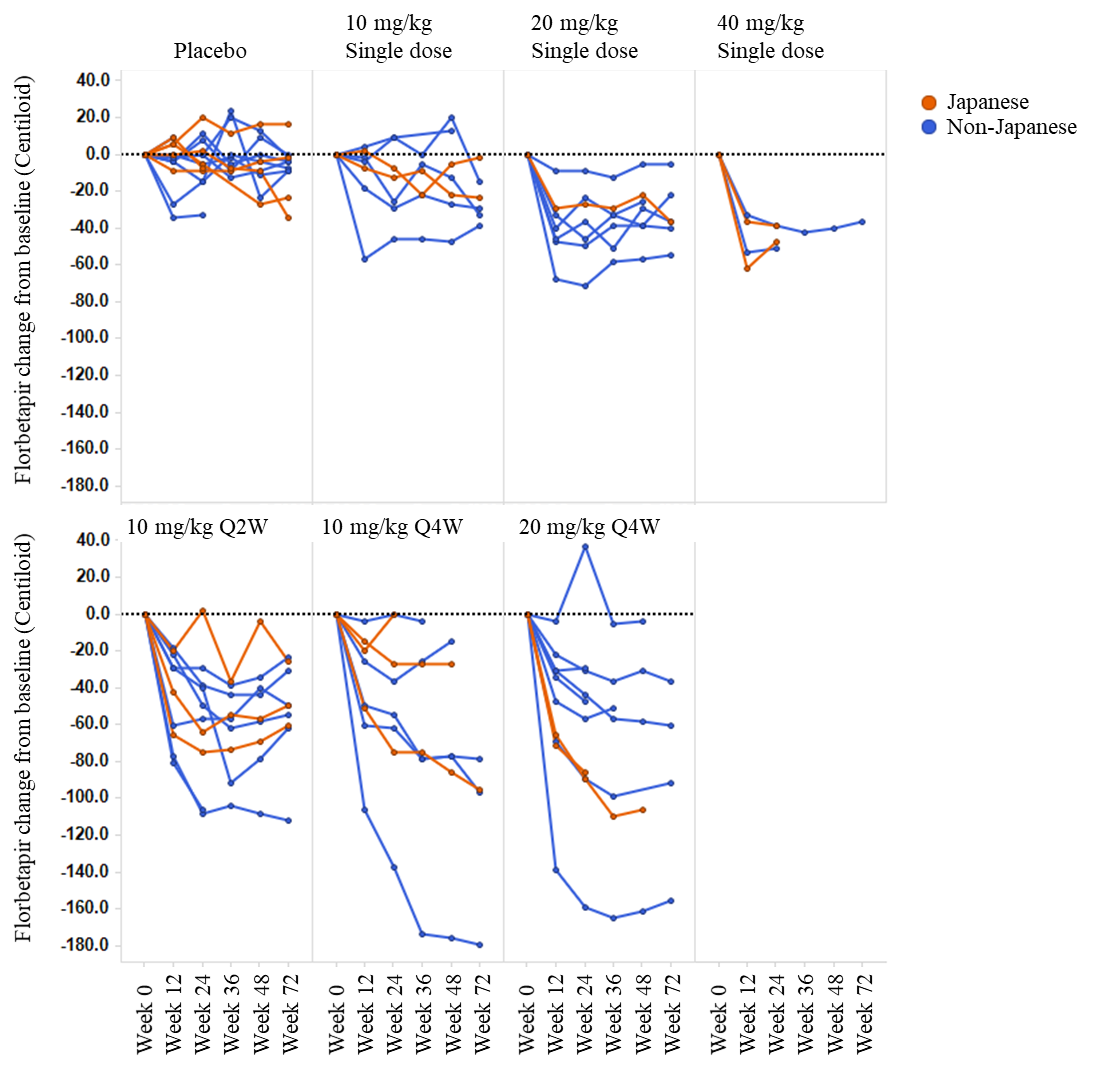
**

**Supplemental Figure 3:** Reduction in cerebral amyloid (Centiloid units) changes from baseline were visually comparable between non-Japanese (blue) and Japanese (red) patients regardless of treatment arm.

Abbreviations: Q2W = every 2 weeks; Q4W = every 4 weeks; SUVr = standardized uptake value ratio.

**Supplemental Table 1:** Summary PK Parameters for Single Dose Cohorts

| **Geometric Mean (CV%)** | | | |
| --- | --- | --- | --- |
| **Serum Donanemab** | | | |
| **Treatment** | **10 mg/kg** | **20 mg/kg** | **40 mg/kg** |
| N | 7 | 7 | 4 |
| C_max_ (ug/mL) | 196 (17) | 413 (17) | 910 (15) |
| t_max_^†^ (h) | 2.07 (1.28 **–** 3.20) | 2.07 (1.78 **–** 3.02) | 2.90 (2.47 **–** 3.22) |
| t_1/2_^‡^ (days) | 10.3 (5.4 – 14.5) | 9.3 (5.6 – 16.2) | 8.3 (6.8 – 11.3) |
| CL (L/h) | 0.0264 (45) | 0.0238 (15) | 0.0221 (14) |
| V_z_ (L) | 9.40 (57) | 7.70 (46) | 6.36 (23) |
| AUC(0-t_last_) (ug.h/mL) | 25700 (20) | 59400 (19) | 110000 (32) |
| AUC(0-∞) (ug.h/mL) | 26200 (19) | 60500 (18) | 112000 (30) |

^†^Median (range).

^‡^Geometric mean (range).

Abbreviations: AUC = area under the concentration versus time curve; AUC(0-∞) = AUC from zero to infinity; AUC(0-tlast) = AUC from time zero to time t, where t is the last time point with a measurable concentration; CL = total body clearance of drug calculated after intravenous administration; C_max_ = maximum observed drug concentration; CV = coefficient of variation; N = number of participants; PK = pharmacokinetic; SAD = single ascending dose; t_1/2_ = half-life associated with the terminal rate constant in noncompartmental analysis; tmax = time of Cmax; V_z_ = volume of distribution during the terminal phase.

**Supplemental Table 2:** Summary PK Parameters for Multiple Dose Cohorts (Day 1)

| **Geometric Mean (CV%)** | | | | |
| --- | --- | --- | --- | --- |
| **Serum Donanemab (Day 1 Profile)** | | | | |
| **Treatment** | **10 mg/kg-Q2W**^§^ | **10 mg/kg-Q2W**^¶^ | **10 mg/kg-Q4W** | **20 mg/kg-Q4W** |
| N | 10 | 9 | 8 | 10 |
| C_max_ (ug/mL) | 179 (82) | 223 (19) | 252 (31) | 564 (26) |
| t_max_^†^ (h) | 1.44 (1.12 **–** 3.05) | 1.43 (1.12 **–** 3.05) | 3.10 (1.30 **–** 3.43) | 2.66 (1.90 **–** 3.32) |
| t_1/2_^‡^ (days) | 5.4 (4.7**–**7.1) | 5.3 (4.7**–**7.1) | 8.1 (5.4 **–** 12.9) | 8.3 (6.2 **–** 14.3) |
| CL (L/h) | 0.0325 (89) | 0.0257 (21) | 0.0182 (23) | 0.0230 (19) |
| V_z_ (L) | 6.08 (97) | 4.74 (22) | 5.12 (15) | 6.64 (23) |
| AUC_Ƭ,day 1_ (ug.h/mL) | 18400 (85) | 23000 (22) | 32900 (35) | 58900 (22) |

^†^Median (range).

^‡^Geometric mean (range).

^§^With Participant 1503 Day 1 profile.

^¶^Without Participant 1503 Day 1 profile.

Abbreviations: AUC = area under the concentration versus time curve; AUC_Ƭ,day 1_ = area under the concentration versus time curve during one dosing interval at day 1, CL = total body clearance of drug calculated after intravenous administration; C_max_ = maximum observed drug concentration; CV = coefficient of variation; MAD = multiple ascending dose; N = number of participants; PK = pharmacokinetic; Q2W = every 2 weeks; Q4W = every 4 weeks; t_1/2_ = half-life associated with the terminal rate constant in noncompartmental analysis; tmax = time of C_max_; V_z_ = volume of distribution during the terminal phase.


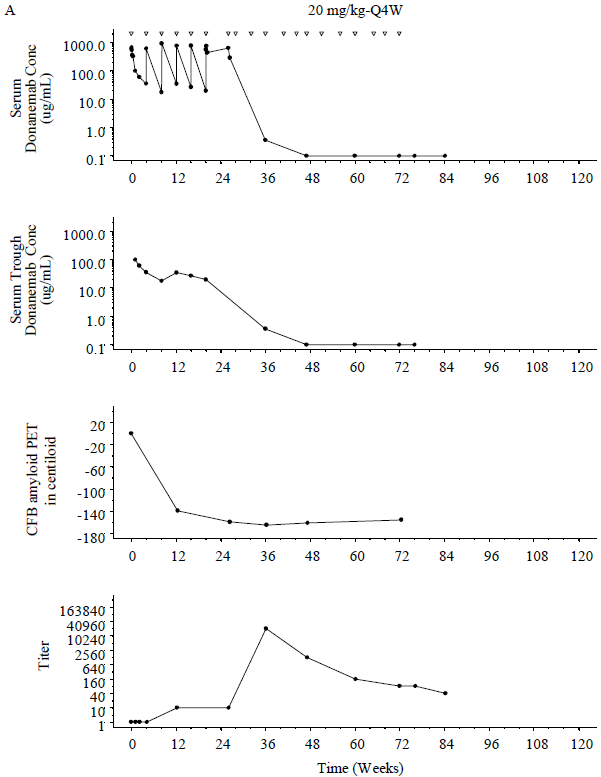
 **
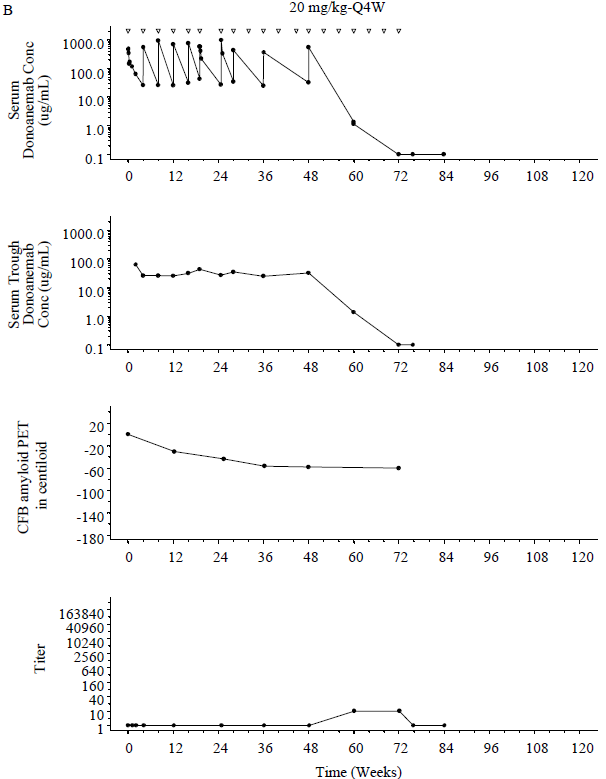
**

Supplemental Figure 4. Donanemab serum concentrations, donanemab trough serum concentrations, CFB amyloid PET in centroid, and ADA Titer over time in weeks for A) an individual selected participant with high titer and lower trough serum concentration and B) an individual selected participant with low titer and lower trough serum concentration. Doses are indicated by open triangles.

Note: BQL samples assigned 0.1 µg/mL values.

Abbreviations: ADA = antidrug antibody; BQL = below the limit of quantification; CFB = change from baseline; Conc = concentration; PET = positron emission tomography; Q4W = every 4 weeks.

**
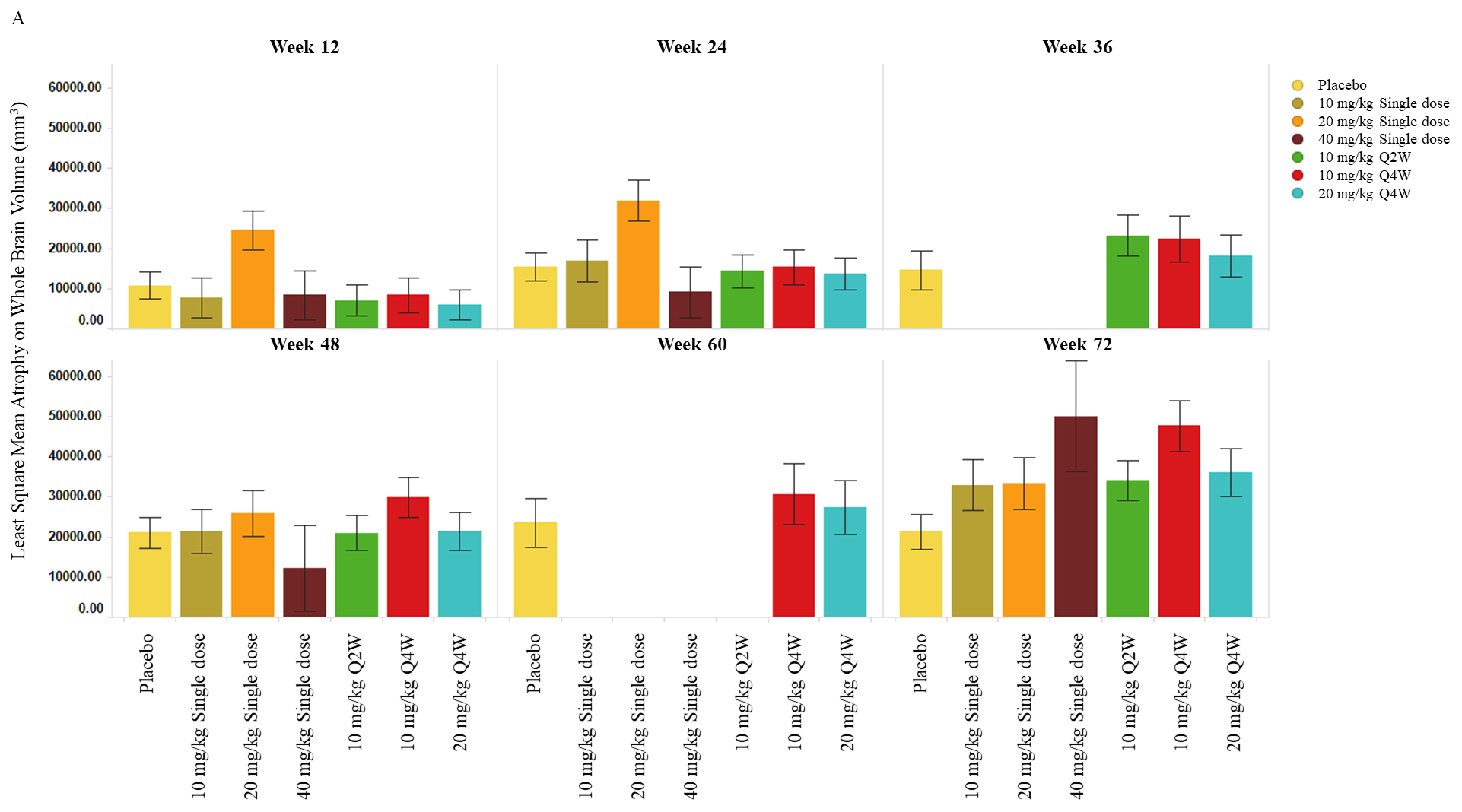
**

**
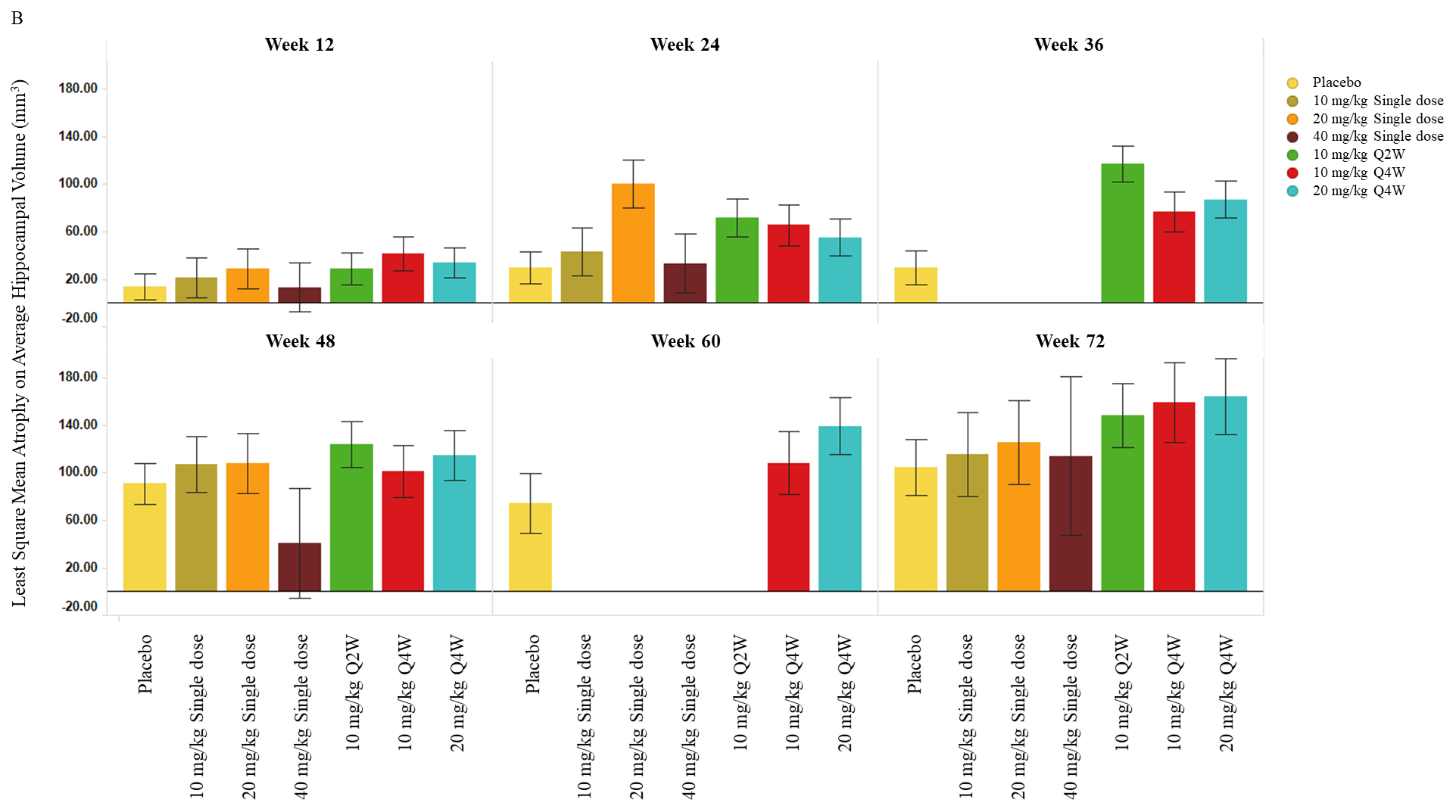
**

**
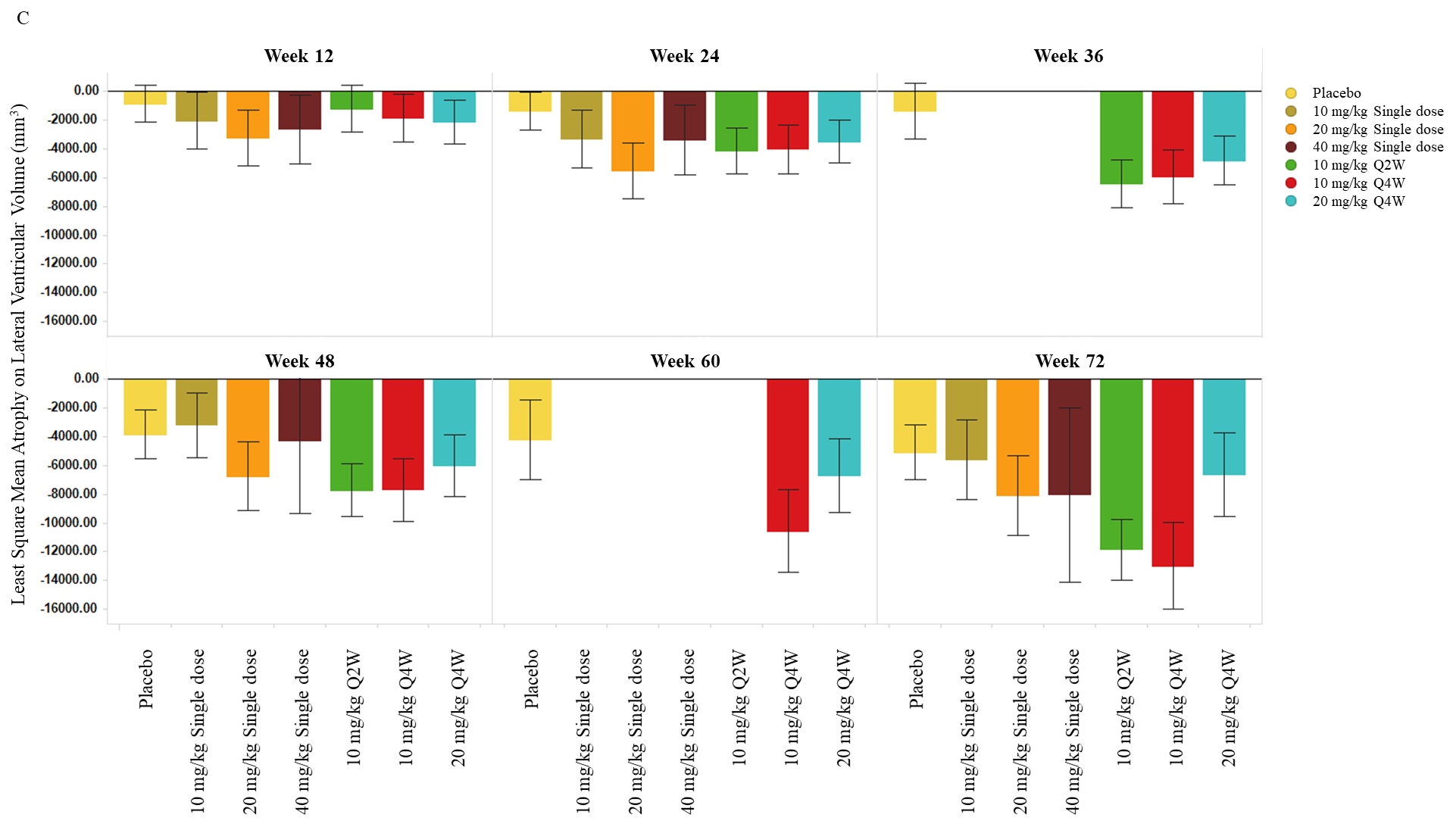
**

**Supplemental Figure 5.** Least squares mean atrophy on A) whole brain volume, B) average hippocampal volume, and C) lateral ventricle volume (mm^3^) per visit per study intervention group.

Abbreviations: Q2W = every 2 weeks; Q4W = every 4 weeks.
